# Supplementary figures and images for: Transcriptomic and Physiological Response of Durum Wheat Grain to Short-Term Heat Stress during Early Grain Filling
Source: Plants (Basel). 2021 Dec 25;11(1):59. doi: 10.3390/plants11010059 (PMC8747107; doi:10.3390/plants11010059)

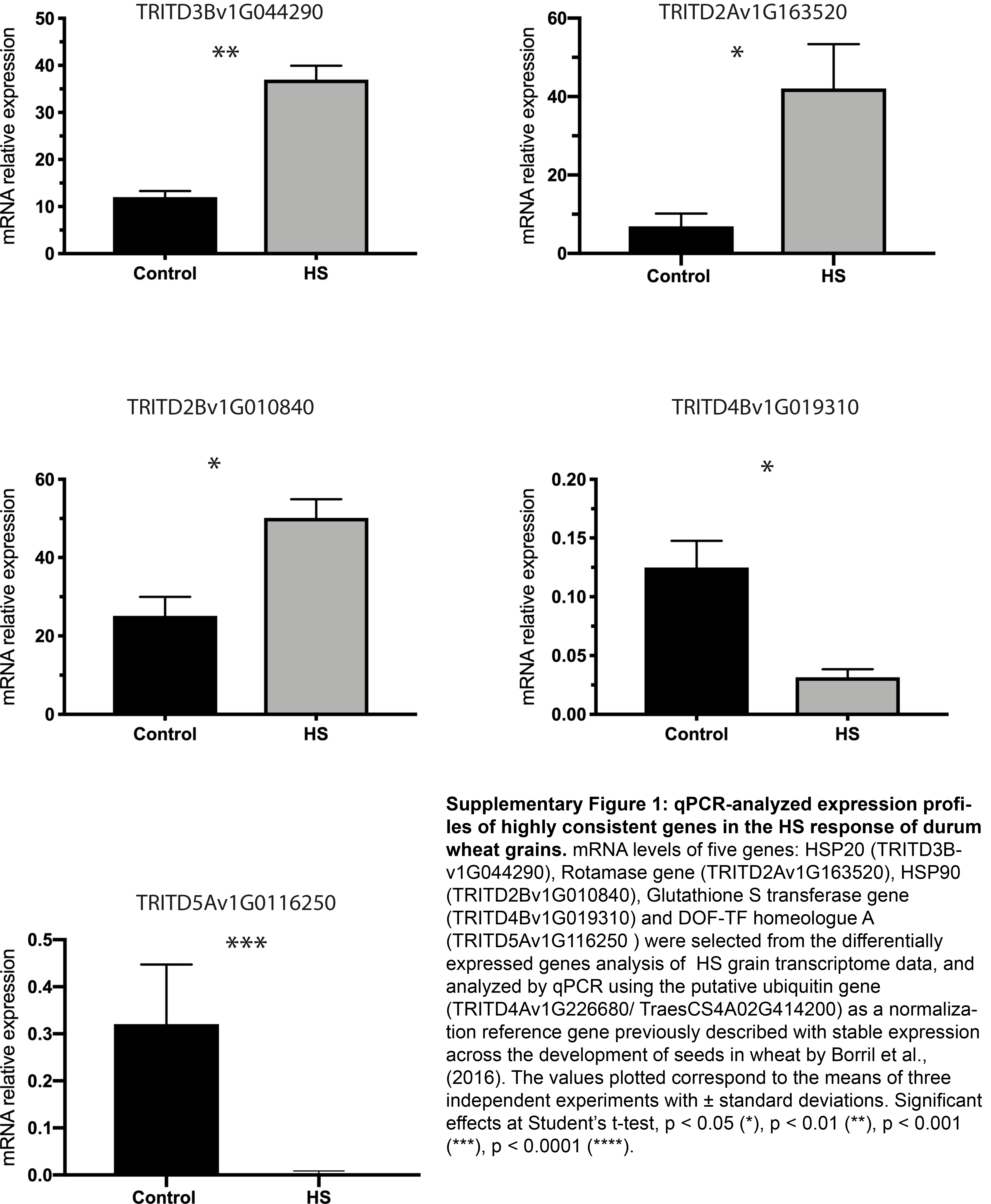

Supplement: Supplementary file 1 [file plants-11-00059-s001.zip › Supplementary Figure S1.jpg]
